# Psychological predictors of work addiction and problematic internet use among physicians: the role of anxiety, well-being, and work-life imbalance

**DOI:** 10.1192/j.eurpsy.2026.10209

**Published:** 2026-07-31

**Authors:** T. T. Simonyi, K. Kostyál, O. Csabai, I. K. Pribék, K. M. Hegedűs, A. Kukla, D. Varga, B. Paksi, Z. Demetrovics, P. Z. Álmos

**Affiliations:** 1 Albert Szent-Györgyi Medical School, Szeged; 2 Institute of Education, ELTE Eötvös Loránd University, Budapest, Hungary; 3 Flinders University Institute for Mental Health and Wellbeing, College of Education, Psychology and Social Work, Flinders University, Bedford Park, SA, Australia; 4 Institute of Psychology, ELTE Eötvös Loránd University, Budapest, Hungary; 5 Centre of Excellence in Responsible Gaming, University of Gibraltar, Gibraltar, Gibraltar

## Abstract

**Introduction:**

Behavioural addictions are increasingly prevalent among medical professionals. These phenomena are becoming more common among doctors worldwide.

**Objectives:**

The aim of this study is to examine the prevalence of work addiction behaviour and problematic internet use among Hungarian physicians and to explore associations with anxiety, well-being, and work-life balance.

**Methods:**

This study uses a cross-sectional survey design involving anonymous questionnaires distributed to 8000 Hungarian physicians, dentists, and clinical psychologists. The measurement tools used were Bergen Work Addiction Scale, Problematic Internet Use Questionnaire, State-Trait Anxiety Inventory, WHO-5 Well-Being Index, and Work-Family Conflict Scale. Descriptive statistics, binary and linear regression, t-test, chi-square test, and frequency analyses were used in the data analysis.

**Results:**

Of the 2826 initiated surveys, 1380 were completed, resulting in a response rate of 17.23%. Among respondents, 32% (n=438) identified as male and 68% (n=941) as female, with ages ranging from 25 to 89 years (M=51.3).

A total 15.3% (n=211) of participants belong to the risk group of work addiction. The risk of work addiction was most common among general practitioners (18.5%), followed by infant and pediatric medicine (10.4%) and anesthesiology and intensive care (5.7%). State anxiety (χ2(1) = 58.243; *p* < 0.001), trait anxiety (χ2(1) = 66.403; *p* < 0.001) and work-family conflict (χ2(1) = 97.303; *p* < 0.001) significant predictors of work addiction.

Considering problematic internet use, 18.3% (n=252) of physicians belong to the high risk group. With regard to problematic internet use subscales, only the control disorder subscale showed a gender difference, with women scoring higher (t(1350) = 0.150; *p* = 0.04). Younger age groups are more likely to belong to the risk group (χ2(4) = 151.23; *p* < 0.001). Well-being was a significant negative predictor of problematic internet use (F(1,1344) = 102.6; *p* < 0.001; R² = 0.071; B = -0.52).

**Conclusions:**

The study highlights the prevalence of work addiction and problematic internet use among Hungarian doctors. State and trait anxiety, as well as work-family conflict, are significant predictors of work addiction: the higher the level of anxiety and the more difficult the work-life balance, the greater the risk of work addictive behaviour. Control disorder subscale is more common among women, they find it more difficult to regulate their internet use. Problematic internet use is more common among younger doctors. Better mental health may be a protective factor, although well-being only partially explains problems related to internet use, suggesting that other factors are also involved. The results highlight the role of several psychological and demographic variables that should be considered when identifying risk groups and planning targeted interventions.

**Disclosure of Interest:**

T. Simonyi Grant / Research support from: This abstract is financially supported by Hetényi Géza grant (number: 5S 764 (A202)) from SZAOK Faculty Research Fund., K. Kostyál: None Declared, O. Csabai: None Declared, I. Pribék: None Declared, K. Hegedűs: None Declared, A. Kukla: None Declared, D. Varga: None Declared, B. Paksi: None Declared, Z. Demetrovics: None Declared, P. Álmos: None Declared

